# A stress-responsive NAC transcription factor SNAC3 confers heat and drought tolerance through modulation of reactive oxygen species in rice

**DOI:** 10.1093/jxb/erv386

**Published:** 2015-08-10

**Authors:** Yujie Fang, Kaifeng Liao, Hao Du, Yan Xu, Huazhi Song, Xianghua Li, Lizhong Xiong

**Affiliations:** National Key Laboratory of Crop Genetic Improvement, National Center of Plant Gene Research (Wuhan), Huazhong Agricultural University, Wuhan 430070, China

**Keywords:** Abiotic stress, NAC, *Oryza sativa*, ROS, transcription factors.

## Abstract

Adverse environmental conditions such as high temperature and drought stress greatly limit the growth and production of crops worldwide. Several NAC (NAM, ATAF1/2, and CUC2) proteins have been documented as important regulators in stress responses, but the molecular mechanisms are largely unknown. Here, a stress-responsive NAC gene, *SNAC3* (ONAC003, LOC_Os01g09550), conferring drought and heat tolerance in rice is reported. *SNAC3* was ubiquitously expressed and its transcript level was induced by drought, high temperature, salinity stress, and abscisic acid (ABA) treatment. Overexpression (OE) of *SNAC3* in rice resulted in enhanced tolerance to high temperature, drought, and oxidative stress caused by methyl viologen (MV), whereas suppression of *SNAC3* by RNAi resulted in increased sensitivity to these stresses. The *SNAC3*-OE transgenic plants exhibited significantly lower levels of H_2_O_2_, malondiadehyde (MDA), and relative electrolyte leakage than the wild-type control under heat stress conditions, implying that *SNAC3* may confer stress tolerance by modulating reactive oxygen species (ROS) homeostasis. Quantitative PCR experiments showed that the expression of a large number of ROS-scavenging genes was dramatically increased in the *SNAC3*-OE plants, but significantly decreased in the *SNAC3*-RNAi transgenic plants. Five ROS-associated genes which were up-regulated in *SNAC3*-OE plants showed co-expression patterns with *SNAC3*, and three of the co-expressed ROS-associated enzyme genes were verified to be direct target genes of SNAC3. These results suggest that SNAC3 plays important roles in stress responses, and it is likely to be useful for engineering crops with improved tolerance to heat and drought stress.

## Highlight

A novel NAC transcription factor regulates tolerance of rice to multiple abiotic stresses through directly targeting the genes related to reactive oxygen species homeostasis.

## Introduction

Plants frequently encounter diverse environmental cues which restrict their growth and development. Among the adverse external stimuli, extreme temperature and water deficit are two major factors that plants frequently confront. To compensate for their sessile lifestyle, plants have evolved a series of sophisticated but efficient strategies to cope with stress conditions. Among these strategies, plants respond to the stresses through stress-specific signalling pathways, which ultimately lead to morphological, physiological, and biochemical changes to adapt to unfavourable environmental conditions. Plants perceive the external signals through sensors, and trigger changes in expression of numerous stress-responsive genes and the synthesis of diverse functional proteins to enable them to survive. Based on the biological functions, genes responsive to the stresses mainly include the functional genes, which encode functional proteins or other products to protect plant cells directly from damage, and the regulatory genes whose products can regulate signal perception and transduction, and the expression of downstream genes under stress conditions (Hirayama and Shinozaki, 2010). Various transcription factors function as central regulators and molecular switches for gene expression control in the stress signalling and adaptation networks (Zhang *et al.*, 2011).

Increasing evidence supporting the role of NAC (NAM, ATAF1/2, and CUC2) transcription factors as key regulators in response to abiotic stresses has been reported in recent years (Puranik *et al.*, 2012). NAC transcription factors comprise one of the largest gene families, which to date are only found in plants. NAC proteins are identified by a highly conserved DNA-binding domain, which is termed the NAC domain, in the N-terminal region, whereas the transcription regulatory region in the C-terminal of NAC proteins is usually diversified in both amino acid composition and length. NAC genes have been reported to be implicated in organ development and boundary maintenance, cell division, secondary wall synthesis, senescence, iron homeostasis, and defence agianst pathogens, and they also act as master regulators in abiotic stress responses (Takada *et al.*, 2001; Guo and Gan, 2006; Kim *et al.*, 2006; Uauy *et al.*, 2006; Zhong *et al.*, 2006; Puranik *et al.*, 2012). The *RD26* (*RESPONSIVE TO DEHYDRATION 26*) gene was the first NAC gene identified as a regulator in mediating cross-talk between abscisic acid (ABA) and jasmonate (JA) signalling during stress responses in *Arabidopsis* (Fujita *et al.*, 2004; Shinozaki and Yamaguchi-Shinozaki, 2007). Overexpressing ANAC019, ANAC055, or ANAC072, which were induced by drought, salt, and ABA, conferred drought tolerance in transgenic *Arabidopsis* (Tran *et al.*, 2004). Tran *et al.* (2004) identified the consensus NACRS [NAC recognition site, CGT(G/A)] and CDBS (core DNA-binding sequence, CACG) in the promoter region of the *Arabidopsis ERD1* (*EARLY RESPONSIVE TO DEHYDRATION 1*) gene. The recognition sequence for NAC factors might be conserved in plants, since quite a few NAC factors can bind to this NACRS (Fujita *et al.*, 2004; Hu *et al.*, 2006, 2008; Liu *et al.*, 2010).

Reactive oxygen species (ROS) are byproducts mainly from aerobic respiration, and were initially considered as toxic molecules which caused oxidative damage to DNA, protein, and membrane lipids in plant cells. Recent studies have demonstrated that ROS also act as important signalling molecules in the complex networks of stress responses (Bechtold *et al.*, 2013; Baxter *et al.*, 2014; Shi *et al.*, 2014). Plants have developed complicated scavenging and regulation pathways to monitor tightly the ROS redox homeostasis in order to avoid the excessive accumulation of ROS in cells. Emerging evidence supports that NAC transcription factors also participate in the regulation of ROS metabolism. An *Arabidopsis* NAC transcription factor, NTL4, which was induced by drought and heat, triggered ROS production by directly binding to *Atrboh* gene promoters, resulting in leaf senescence (Lee *et al.*, 2012). Another NAC factor, JUB1, was described as a major longevity regulator in *Arabidopsis* (Wu *et al.*, 2012). *JUB1* was H_2_O_2_-responsive and could bind to the *DREB2A* promoter. Overexpressing *JUB1* promoted leaf longevity and tolerance to salt and heat stress conditions (Shahnejat-Bushehri *et al.*, 2012; Wu *et al.*, 2012). A membrane-associated NAC protein, ANAC013, was shown to bind and transactivate the mitochondrial dysfunction motif (MDM) to mediate mitochondrial retrograde regulation (MRR)-induced gene expression, and conferred increased tolerance to methyl viologen (MV) and rotenone-induced oxidative stress in transgenic *Arabidopsis* plants (De Clerq *et al.*, 2013).

As one of the most important crops worldwide, rice provides staple food for more than half of the world’s population. In facing climate changes and increasing global population, it is an urgent task to exploit stress resistance and yield stability of rice under adverse environmental conditions. In the past few decades, various stress-responsive transcription factors including NACs have been explored for engineering rice for improved stress tolerance through transgenic approaches (Sacar *et al.*, 2013). *SNAC1* overexpressors exhibited better performance under drought and salt stress conditions at the vegetative stage, and the transgenic plants also exhibited higher seed production under drought conditions at the reproductive stage (Hu *et al.*, 2006). Overexpression of *SNAC1*, *OsNAC10*, and *OsNAC5* driven by a root-specific promoter *RCc3* enlarged the root diameter in rice, and therefore conferred increased drought resistance and grain yield under field drought conditions (Ishizaki *et al.*, 2006; D.H. Jeong *et al.*, 2010; J.S. Jeong *et al.*, 2010, 2013).

In this study, another NAC transcription factor gene, *SNAC3*, which was responsive to various abiotic stresses, was characterized. The *SNAC3* overexpression (OE) transgenic plants enhanced heat, drought, and oxidative tolerance in rice, whereas repressing *SNAC3* weakened heat, osmotic, and oxidative tolerance. The data indicated that *SNAC3* regulates the heat stress response by adjusting the redox homeostasis state through controlling the expression of ROS-associated enzyme genes. It was also found that the *SNAC3*-mediated stress responses may be ABA-independent, which is different from many previous reports of stress-responsive NAC genes in rice. The findings indicate that SNAC3 is a crucial regulator in heat and drought stress responses, and has great potential in engineering crops with enhanced heat and drought tolerance.

## Materials and methods

### Plant materials and stress treatments

The *japonica* rice (*Oryza sativa*) cultivar (Zhonghua 11, ZH11) was used as a transformation recipient in this study. To detect the transcript level of *SNAC3* under various stresses and phytohormone treatments, ZH11 plants were planted in the greenhouse with a 14h light/10h dark cycle. Four-leaf stage seedlings were subjected to a variety of abiotic stresses including drought (stopping water supply), salt (irrigation with 200mM NaCl solution), heat (transferring the seedlings to a 42 °C growth chamber), cold (exposing the plants to 4 °C), submergence (covering the plants completely with water), wounding (cutting the leaves into pieces and floating them on water at room temperature under continuous light conditions), H_2_O_2_ treatment [irrigation with 1% (v/v) H_2_O_2_ solution], and ABA treatment (spraying 100 μM ABA on the leaves), followed by sampling at the designated times.

*SNAC3*-OE and RNAi transgenic plants were selected by germinating seeds on Murashige and Skoog (MS) medium containing 50mg l^–1^ hygromycin. Wild-type (WT) seeds were germinated on normal MS medium. For heat stress treatment at the seedling stage, transgenic plants and WT plants were grown in the same barrel in a split (half-and-half) manner under normal growth conditions. Seedlings at the four- to five-leaf stage were subjected to heat stress treatment by transferring the plants to a 42 °C growth chamber. After heat stress treatment for 1–2 d, the seedlings were allowed to recover in normal growth conditions for 1 week. For drought stress treatment at the vegetative stage, transgenic plants and WT plants were grown in the same barrel in a split manner under normal growth conditions until the four- to five-leaf stage. The water supply was withheld to cause drought stress until the WT leaves became completely wilted. After recovery by rewatering for a week, the survival rates were recorded and the plants were photographed. Drought stress treatment at the reproductive stage was executed in a refined paddy field facilitated with a movable rain-off shelter by stopping the irrigation at the booting stage followed by re-watering after flowering. To evaluate the tolerance of rice seedlings to mannitol stress, MV, and ABA, germinated seeds were transferred to MS medium containing 120mM mannitol, 2 μM MV, and 3 μM ABA, respectively. After growing on the medium for 7–10 d, the shoot lengths were measured to evaluate the tolerance.

### Plasmid construction and transformation of rice

To create a *SNAC3*-OE construct, the coding region of *SNAC3* was obtained from rice total RNA by reverse transcription–PCR (RT–PCR). The full-length cDNA of *SNAC3* was inserted into the pU1301 vector downstream of the *Ubiquitin* promoter. To generate a dsRNAi construct of *SNAC3*, a 334bp *SNAC3*-specific fragment was introduced into the pDS1301 vector (Yuan *et al.*, 2007). To investigate the expression pattern of *SNAC3*, a genomic DNA sequence from the promoter region (2536 to 303bp upstream of the predicted ATG codon of the ORF) of *SNAC3* was inserted into the DX2181 vector to drive the *GUS* (β-glucuronidase) reporter gene. The constructs were transformed into the *japonica* rice cv. ZH11 using the *Agrobacterium*-mediated transformation method (Lin and Zhang, 2005). The primers used in this study are listed in Supplementary Table S1 available at *JXB* online.

### RNA isolation and analysis of its expression level

Total RNA was extracted from rice leaves using Trizol reagent (Invitrogen, Carlsbad, CA, USA). The first-strand cDNA was synthesized using Superscript III reverse transcriptase (Invitrogen) according to the manufacturer’s instructions. Quantitative RT–PCR was conducted on a 7500 Real-time PCR System (Applied Biosystems, Foster City, CA, USA) using SYBR Premix Ex Taq (TaKaRa, Dalian, China) according to the manufacturer’s protocol. The rice *Ubiquitin* gene was used as the internal control. The relative expression level was determined as described previously (Livak and Schmittgen, 2001). For the promoter–GUS reporter assay, various tissues and organs of the pDX2181-*SNAC3*pro::GUS transgenic plants were collected for GUS histochemical staining essentially as described previously (Ning *et al.*, 2010). Quantification of GUS activity was carried out according to the method described previously (Huang *et al.*, 2007).

### Determination of stress-associated physiological indicators

For water loss rate measurement, detached leaves from *SNAC3*-OE and the WT plants were weighed at the indicated times. The cell membrane permeability was evaluated by electrolyte leakage measured by the relative conductance method (Bajji *et al.*, 2002). For estimation of the degree of membrane lipid peroxidation in cells, the MDA (malondialdehyde) content was determined as described previously (Du *et al.*, 2010). H_2_O_2_ was visually detected by staining the leaves of the *SNAC3*-OE and the WT plants with 3,3′-diaminobenzidine (DAB) as described previously (Ning *et al.*, 2010). The reddish brown coloration produced by DAB staining for H_2_O_2_ was recorded. Quantification of H_2_O_2_ was performed using a kit (Invitrogen) by following the manufacturer’s instructions. For the quantification of ABA content, leaves from the four- to five-leaf-stage WT and *SNAC3*-OE seedlings before and after stress treatment were sampled and prepared following a modified crude extraction procedure (Pan *et al.*, 2008). ABA content determination was executed by an Applied Biosystems 4000Q-TRAR LC-MS system with stable isotope-labelled ABA as a standard from OlChemIm according to an ultra-fast liquid chromatography-electrospray ionization tandem mass spectrometry (UFLC-ESI-MS/MS)-based method described preciously (Ding *et al.*, 2008; Liu *et al.*, 2012).

### Biochemical assay in yeast

The yeast one-hybrid assay was carried out using the Matchmaker one-hybrid system following the manual (Clontech, Palo Alto, CA, USA). The promoter fragments of potential SNAC3-targeted genes containing the NACRS and NDBS *cis*-elements were amplified and fused upstream of the *HIS3* minimal promoter in the pHIS2 vector to generate reporter constructs. The ORF of SNAC3 obtained from PCR amplification were fused to the GAL4 activation domain in the pGAD7-Rec2 vector (Clontech), and subsequently co-transformed with the reporter constructs described above into the yeast strain Y187. In the meantime, the pGAD-53 and pHIS-53 plasmids were co-transformed as positive controls, while the pGAD-SNAC3 and pHIS2-53 plasmids were co-transformed into Y187 as negative controls. Growth performances of the transformants on the SD/-Leu/-Trp and SD/-Leu/-Trp/-His media containing 30mM 3-aminotriazole (3-AT) were analysed to evaluate the DNA–protein interactions.

### Subcellular localization assays

The full-length cDNA of *SNAC3* was amplified and fused into the pHBT-sGFP destination vector via a restriction enzyme-mediated cloning procedure, and into the pH7WGF2,0 binary vector by GATEWAY recombination reaction (Invitrogen) (Karimi *et al.*, 2007), respectively. The SNAC3-pHBT-sGFP plasmid was transformed into rice protoplasts to perform the transient expression assay as described previously (You *et al.*, 2013). The SNAC3-pH7WGF2,0 plasmid was introduced into *Nicotiana benthamiana* leaves through an *Agrobacterium*-mediated method. For transient expression, an *Agrobacterium* strain (EHA105) containing the SNAC3-pH7WGF2,0 construct was infiltrated at an OD_600_ of 1.0 into the 5- to 6-week-old *N. benthamiana* leaves. After incubation for 12–16 h, the distribution of the fusion protein was captured using a confocal fluorescence microscope (Leica TCS SP2).

### Protein interaction analysis

The yeast two-hybrid assay was performed using the Matchmaker™ Gold Yeast Two-Hybrid system (Clontech). According to previous reports, two amino acids (Gly and Glu) at the N-terminal end of NAC proteins may participate in the formation of NAC protein dimers (Ernst *et al.*, 2004; Jeong *et al.*, 2009). To avoid the interference caused by dimer formation between SNAC3 and other NAC proteins, the coding region without the 108 amino acids at the N-terminus of SNAC3 was amplified and inserted in-frame with GALBD into the pGBKT7 vector to generate the bait construct, and the yeast two-hybrid library was constructed using the leaves from the rice seedlings exposed to heat and cold stress conditions. The two-hybrid screening was carried out using yeast mating technology according to the manufacturer’s instructions. To confirm further the proteins which interact with SNAC3 in living plant cells, bimolecular fluorescence complementation (BiFC) assay was performed in a rice protoplast system as described preciously (Tang *et al.*, 2012).

## Results

### Identification and expression features of *SNAC3*

In the systematic analysis of the NAC family in rice, a significant portion of the family genes were found to be responsive to various abiotic stresses (Fang *et al.*, 2008), and one of them, designated as *SNAC3* or *ONAC003* (LOC_Os01g09550), which belongs to subfamily II, including the closest *Arabidopsis* homologue At4g29230 with unknown function, was subjected to further functional characterization in this study. *SNAC3* is located on chromosome 1 with four predicted differentially spliced transcripts (TIGR, http://rice.plantbiology.msu.edu/). Compared with the second longest transcript (LOC_Os01g09550.2), the longest transcript (LOC_Os01g09550.1) encoded a peptide with 52 additional amino acids at the N-terminus. However, only the full-length cDNA corresponding to the second longest transcript was isolated, and no EST was found for the extended N-terminal region for the predicted longest transcripts. To verify this, a pair of specific primers were designed to amplify the predicted extended N-terminal region by using rice cDNA (genomic DNA as the control) as the PCR template. No amplification was detected in the PCR of the cDNA template (Supplementary Fig. S1 at *JXB* online), indicating that LOC_Os01g09550.1 is not a transcript of *SNAC3*. Therefore, the following experiments were designed based on LOC_Os01g09550.2.

**Fig. 1.**
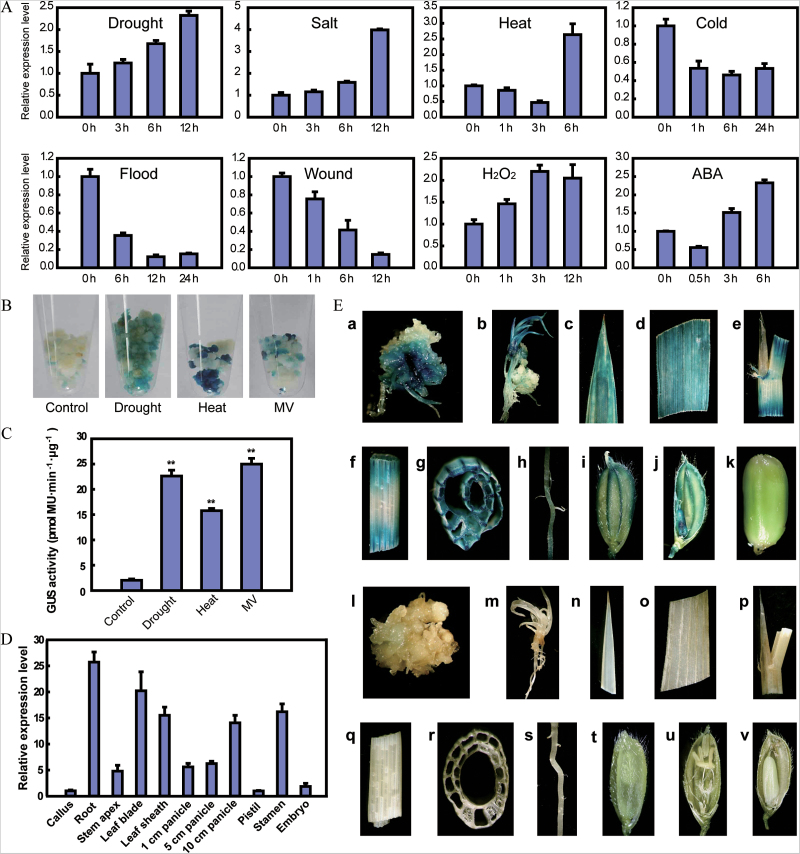
Expression pattern analysis of *SNAC3*. (A) Expression level of *SNAC3* under various abiotic stresses and ABA treatment. Four-leaf stage seedlings were subjected to drought, salt (200 mmol l^–1^ NaCl), heat (42 °C), cold (4 °C), flood, wounding, H_2_O_2_ (1% H_2_O_2_), and ABA treatment (100 mmol l^–1^ ABA). The relative expression level of *SNAC3* was detected by qPCR at the indicated times. Error bars indicated the SE based on three replicates. (B) Expression pattern of the GUS reporter gene driven by the *SNAC3* promoter in transgenic resistant callus under normal conditions (control), drought, heat, and MV stress. (C) GUS activity quantification in *P*_*SNAC3*_:*GUS* transgenic resistant callus under normal conditions, drought, heat, and MV stress. GUS activity was defined in picomoles of 4-MU generated per minute per microgram of protein. (D) Detection of *SNAC3* expression in various tissues and organs using qPCR. Error bars indicate the SE based on three technical replicates. (E) Expression pattern under normal conditions. (a–k) GUS staining of tissues and organs from *P*_*SNAC3*_:*GUS* transgenic plants; (l–v) GUS staining of tissues and organs from ZH11 plants. (a and l) callus; (b and m) regenerated seedling; (c and n) leaf tip; (d and o), leaf blade; (e and p) ligule, collar, and auricle; (f and q) leaf sheath; (g and r) cross-section of the leaf sheath; (h and s) root; (i and t) hull; (j and u) stamen and pistil; (k and v) seed.

*SNAC3* showed stress-inducible expression in the microarray analysis (Zhou *et al.*, 2007). To investigate the expression profile of *SNAC3* comprehensively, quantitative real-time RT–PCR (qPCR) was used to detect the transcript abundance of *SNAC3* in four-leaf stage rice seedlings under a variety of abiotic stresses and hormone treatments. It was found that *SNAC3* was induced by drought, high salinity, heat, oxidative stress, and ABA treatment. However, it was suppressed by cold, submergence, and wounding stresses (Fig. 1A). The induction of *SNAC3* under drought, heat, and MV treatment was further confirmed in the GUS staining assay of *P*_*SNAC3*_:*GUS* transgenic callus (Fig. 1B), and the quantification of GUS activity was in agreement with the staining results (Fig. 1C).

A total of 11 representative tissues/organs (callus, root, stem apex, leaf blade, leaf sheath, 1cm panicle, 5cm panicle, 10cm panicle, pistil, stamens, and embryo) were sampled for spatio-temporal expression analysis of *SNAC3*. As shown in Fig. 1D, *SNAC3* was expressed in all of the tissues/organs tested, and the expression level was relatively lower in callus, pistil, and embryo when compared with other tissues/organs. To clarify the expression pattern of *SNAC3*, transgenic rice expressing the *GUS* reporter gene under the control of the *SNAC3* promoter were generated, and GUS staining was performed to detect the expression of the reporter gene. The GUS signal was detected in almost all of the analysed tissues/organs, confirming that *SNAC3* was expressed ubiquitously (Fig. 1E).

### Overexpression of *SNAC3* in rice enhanced heat and drought resistance

To elucidate the biological functions of *SNAC3* in rice, the *SNAC3*-OE vector was constructed and transformed into rice ZH11. qPCR was carried out to detect the *SNAC3* transcript in transgenic plants. The expression level of *SNAC3* was significantly higher in the transgenic plants than in the WT (Supplementary Fig. S2 at *JXB* online).

**Fig. 2.**
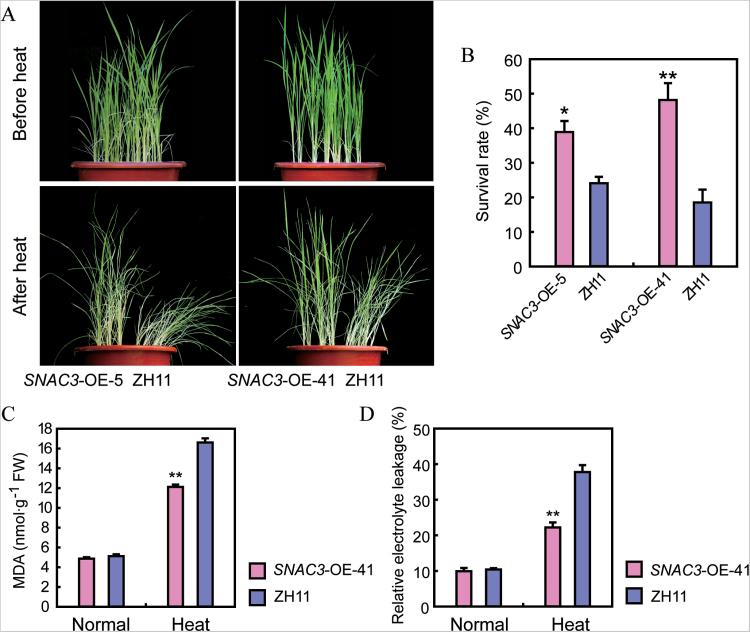
Enhanced heat tolerance of the *SNAC3*-OE transgenic plants at the seedling stage. (A) Phenotype of the *SNAC3*-OE transgenic plants under heat stress conditions. Four-leaf stage plants were subjected to 42 °C heat stress in a growth chamber (14h light/10h dark) for 1–2 d, and then transferred to normal growth conditions. (B) Survival rate of *SNAC3*-OE and ZH11 after heat stress treatment. Data represent the mean ±SE (*n*=3). *P<0.05, *t*-test; **P<0.01, *t*-test. (C) MDA content of *SNAC3*-OE and ZH11 seedlings under normal and heat stress conditions. Data represent the mean ±SE (*n*=3). *P<0.05, *t*-test; **P<0.01, *t*-test. (D) Relative electrolyte leakage of the leaves from *SNAC3*-OE and ZH11 seedlings under normal and heat stress conditions. Data represent the mean ±SE (*n*=3). *P<0.05, *t*-test; **P<0.01, *t*-test.

Since *SNAC3* was strongly induced by high temperature stress, the performance of transgenic rice overexpressing *SNAC3* was first evaluated under heat stress conditions at the vegetative stage. The seedlings at the four-leaf stage were transferred to a 42 °C growth chamber for heat stress treatment. The overexpression plants exhibited leaf rolling and wilted later than the WT during the course of heat stress treatment (Fig. 2A). About 40% of the overexpression plants survived after the heat treatment, whereas the WT only exhibited a 20% survival rate after the same stress treatment (Fig. 2B). One of the OE lines (*SNAC3*-OE-41) was used for further determination of the physiological parameters. Heat stress generally causes membrane lipid peroxidation of plant cells. As a major product of this process, MDA is widely used as an important indicator to evaluate the degree of membrane lipid peroxidation, which reflected the oxidative damage to lipids caused by environmental stress. In addition, cell membrane permeability increases under adverse conditions, and electrolyte leakage has been commonly considered as an indicator to assess cell membrane stability and the extent of the cell membrane damage under stress conditions (Smirnoff, 1993; Agarie *et al.*, 1998). Therefore, the MDA content and electrolyte leakage of *SNAC3*-OE and the WT were measured under heat stress and normal conditions. As shown in Fig. 2, no differences were observed in the MDA content and electrolyte leakage between the *SNAC3*-OE and control plants under normal conditions, while both the MDA content and electrolyte leakage were obviously lower in the *SNAC3*-OE plants compared with the WT plants under heat stress conditions (Fig. 2C, 2D). These results suggested that overexpressing *SNAC3* may cause less extensive membrane lipid peroxidation and improved cell membrane stability under the heat stress treatment, and thus contribute to attenuate the oxidative damage caused by the heat stress.

**Fig. 3.**
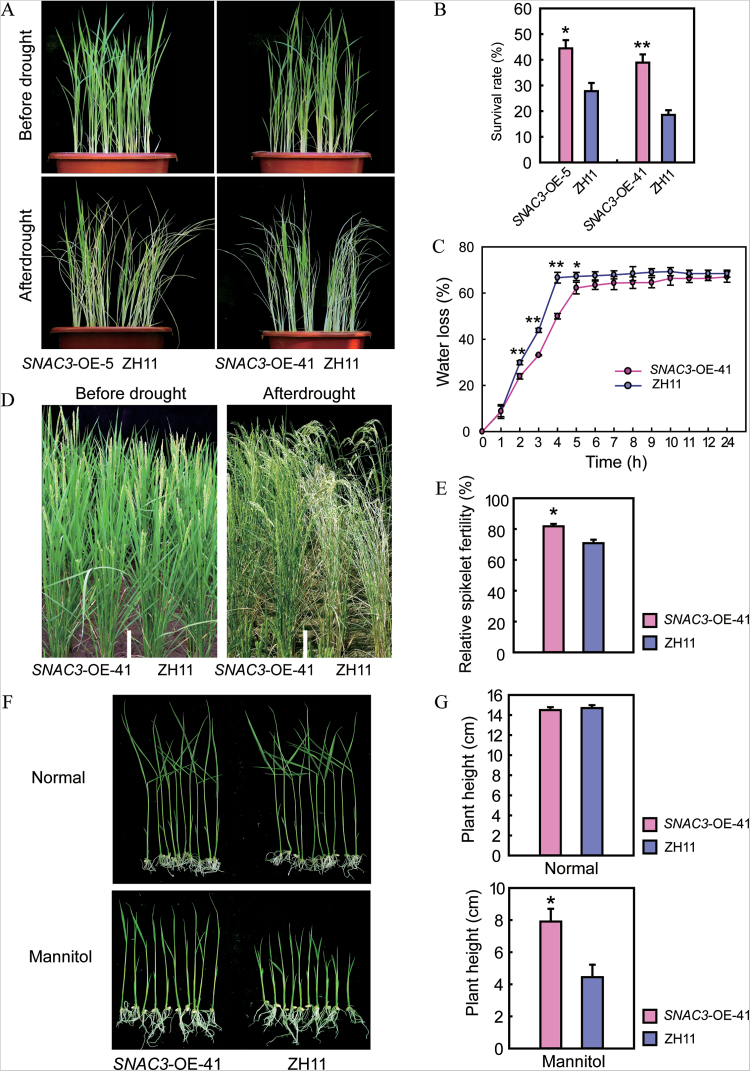
Enhanced drought resistance of the *SNAC3*-OE transgenic plants. (A) Phenotype of the *SNAC3*-OE plants under drought stress conditions at the seedling stage. Four-leaf stage plants were growth without water supply for 10 d, followed by rewatering for 7 d. (B) Survival rates of *SNAC3*-OE and ZH11 after drought stress treatment. Data represent the mean ±SE (*n*=3). *P<0.05, *t*-test; **P<0.01, *t*-test. (C) Water loss rates of detached leaves from the *SNAC3*-OE and ZH11 plants. Data represent the mean ±SE (*n*=3). *P<0.05, *t*-test; **P<0.01, *t*-test. (D) Enhanced drought resistance of *SNAC3*-OE transgenic plants at the reproductive stage. (E) Relative spikelet fertility of *SNAC3*-OE and ZH11 under drought stress treatment at the reproductive stage. Data represent the mean ±SE (*n*=12). *P<0.05, *t*-test. (F) Enhanced tolerance of *SNAC3*-OE plants to osmotic stress conditions. (G) Plant height of *SNAC3*-OE and ZH11 plants under normal and osmotic stress conditions. Data represent the mean ±SE (*n*=12). *P<0.05, *t*-test; **P<0.01, *t*-test.

The *SNAC3*-OE transgenic plants were also tested for drought resistance at both the vegetative and reproductive stages. At the vegetative stage, four-leaf-stage seedlings grown in soil were subjected to drought stress treatment by withholding the water supply for 10 d. After recovery for 7 d, 40–45% of the *SNAC3*-OE plants had recovered, while only 20–25% of the WT plants survived (Fig. 3A, B). These results indicated that *SNAC3*-OE plants exhibited increased drought resistance at the vegetative growth stage. The *SNAC3*-OE-41 line was used for further physiological determination. The water loss rates of detached leaves from the *SNAC3*-OE and WT plants were gauged. In agreement with the phenotype, the detached leaves from *SNAC3*-OE plants lost water more slowly than the WT (Fig. 3C). For drought stress at the reproductive stage, plants were grown in a paddy field equipped with a movable rain-off shelter. Drought stress was initiated at the booting stage by stopping irrigation. *SNAC3*-OE transgenic plants showed later wilting than the WT during the stress treatment (Fig. 3D). After maturation, the relative spikelet fertility of the *SNAC3*-OE plants was significantly (*P*<0.05) higher than that of the WT (Fig. 3E), suggesting that overexpression of *SNAC3* also enhanced drought resistance at the reproductive stage.

The *SNAC3*-OE-41 plants were further tested under osmotic stress conditions. There was no obvious difference between the *SNAC3*-OE and WT plants grown on normal MS medium (Fig. 3F), while the shoot lengths of the *SNAC3*-OE plants were significantly longer than that of the WT plants grown on MS medium containing 120mM mannitol (Fig. 3F, G), indicating that overexpression of *SNAC3* had a positive effect on improving osmotic stress tolerance in rice.

### Suppressing *SNAC3* by RNAi decreased tolerance to heat, drought, and oxidative stresses

**Fig. 4.**
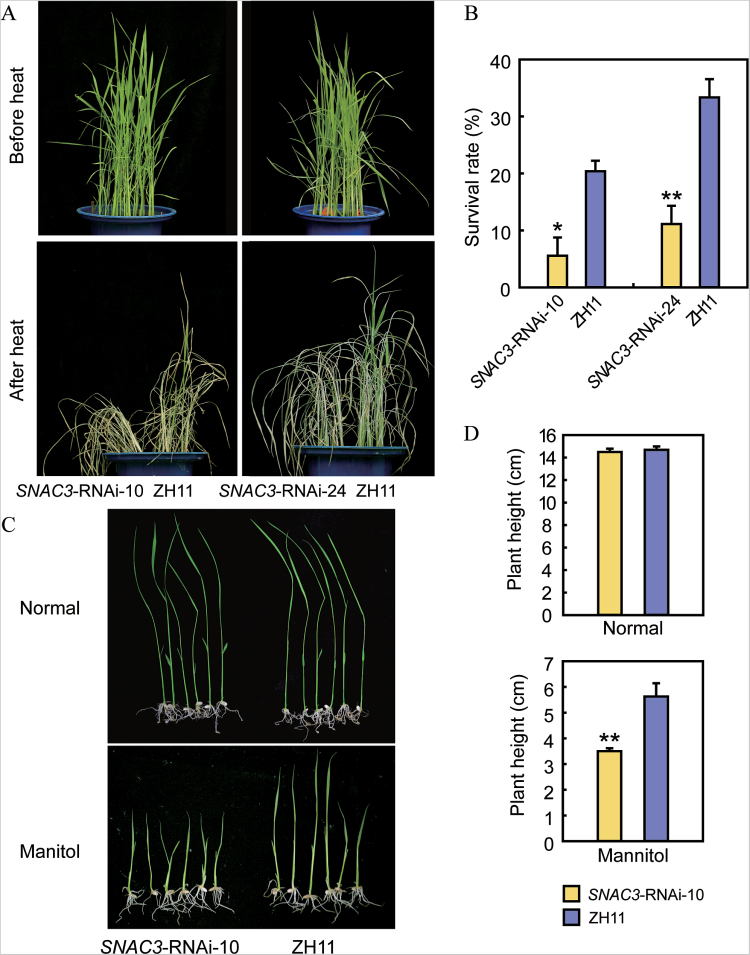
Phenotype of *SNAC3*-RNAi transgenic plants under heat and osmotic stresses. (A) Four-leaf stage plants were subjected to 42 °C heat stress in a growth chamber (14h light/10h dark) for 1–2 d, and then transferred to normal growth conditions. (B) Survival rates of *SNAC3*-RNAi and ZH11 plants after heat stress treatment. Data represent the mean ±SE (*n*=3). *P<0.05, *t*-test; **P<0.01, *t*-test. (C) Enhanced sensitivity of *SNAC3*-RNAi plants to osmotic stress conditions. (D) Plant height of *SNAC3*-RNAi and ZH11 under normal and osmotic stress conditions. Data represent the mean ±SE (*n*=12). **P<0.01, *t*-test.

To validate further the functions of *SNAC3* in rice, an RNAi construct was generated to suppress the endogenous expression of *SNAC3* in transgenic plants. The transcript levels of >30 T_0_ *SNAC3*-RNAi plants were checked by qPCR, and the transcript levels of *SNAC3* in >50% of the transgenic plants were significantly inhibited (Supplementary Fig. S3 at *JXB* online). Four-leaf stage *SNAC3*-RNAi and WT seedlings were subjected to heat stress treatment in a 42 °C growth chamber for 1–2 d. During the course of the stress treatment, the *SNAC3*-RNAi plants wilted earlier than the WT (Fig. 4A). The survival rate of the RNAi seedlings was lower than that of the WT after recovery (Fig. 4B), suggesting that suppressing *SNAC3* weakened the heat tolerance in rice. The responses of the *SNAC3*-RNAi-10 plants to osmotic stress were also tested. In contrast to the WT plants, the RNAi plants showed increased sensitivity to osmotic stress at the post-germination stage (Fig. 4C). After treatment with 120mM mannitol for 7 d, the shoot lengths of the *SNAC3*-RNAi plants were significantly shorter than that of the WT (Fig. 4D). These phenotypes of the *SNAC3*-RNAi plants which were in contrast to the performances of the *SNAC3*-OE plants under the same stress conditions further supported the positive role of *SNAC3* in response to heat, drought, and oxidative stress treatment.

### *SNAC3* participates in the regulation of ROS metabolism and oxidative stress

**Fig. 5.**
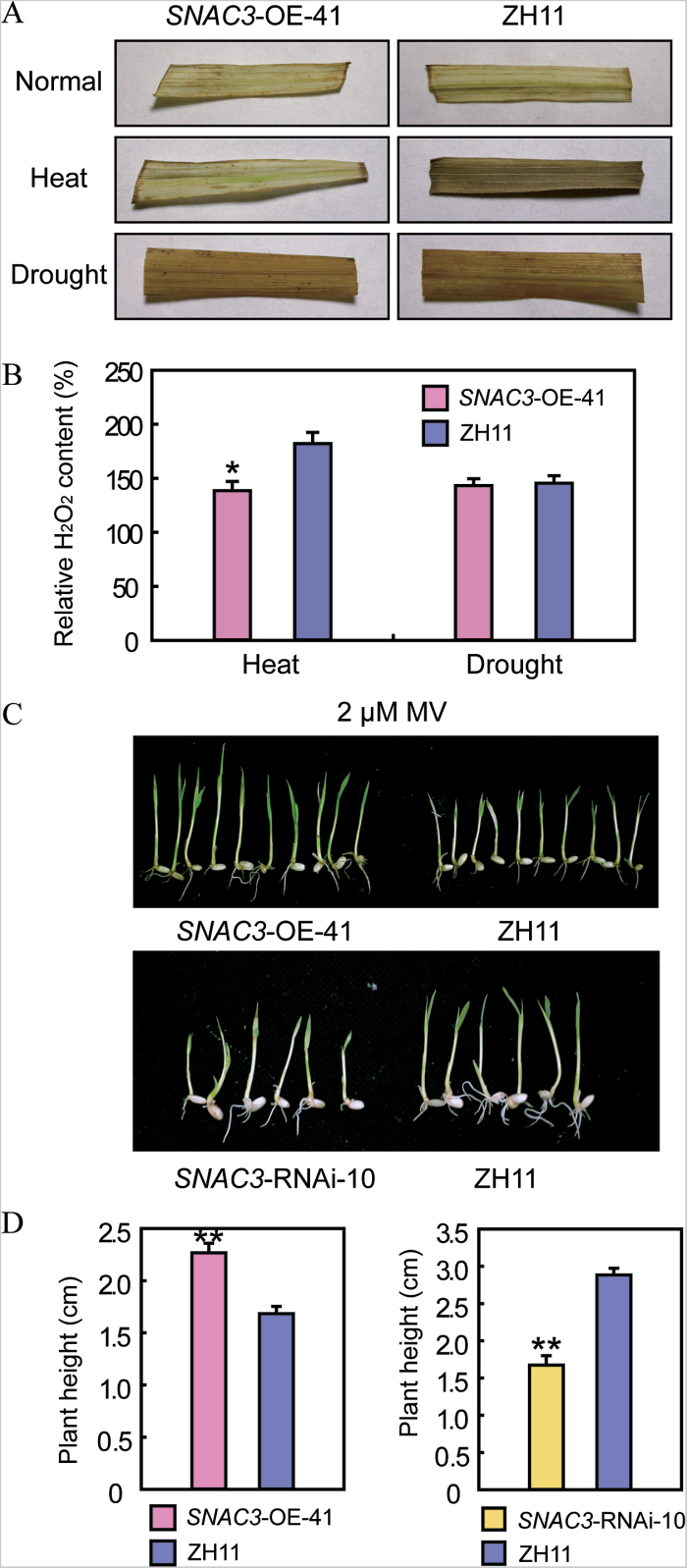
*SNAC3* participated in regulation of ROS metabolism. (A) DAB staining of leaves from *SNAC3*-OE and ZH11 seedlings under normal conditions, and heat and drought stress treatments. (B) Relative H_2_O_2_ content in leaves from *SNAC3*-OE and ZH11 seedlings under heat, drought treatments, and normal conditions. Data are the mean ±SE (*n*=3). *P<0.05, *t*-test. (C) Enhanced tolerance of *SNAC3*-OE plants and enhanced sensitivity of *SNAC3*-RNAi plants to oxidative stress conditions. (D) Plant height of *SNAC3*-OE, *SNAC3*-RNAi, and ZH11 under oxidative stress treatments. Data are the mean ±SE (*n*=12). **P<0.01, *t*-test.

Based on the above results, it was hypothesized that *SNAC3* may be involved in regulating ROS metabolism pathways. Oxidative stress usually results in excessive accumulation of H_2_O_2_. DAB staining analysis indicated that there was no significant difference in H_2_O_2_ accumulation between the *SNAC3*-OE and the WT plants under normal and drought stress conditions. However, the *SNAC3*-OE leaves showed visibly less H_2_O_2_ accumulation under heat stress conditions (Fig. 5A). The quantitative analysis also supports the DAB staining results (Fig. 5B). The potential role of *SNAC3* in oxidative stress tolerance was further examined by using a well-known inducer of oxidative stress, MV (paraquat). Germinated seeds of *SNAC3*-OE, *SNAC3*-RNAi, and the WT with similar vigour were placed on MS medium containing 2 μΜ MV. After 7 d, the *SNAC3*-OE plants exhibited less growth inhibition and etiolation compared with the WT plants, while the RNAi plants showed more severe growth inhibition and etiolation than the WT (Fig. 5C, D). The results suggested that *SNAC3* may positively regulate oxidative stress tolerance.

### The expression of ROS scavenging-related genes was altered significantly in the *SNAC3*-OE and RNAi rice

**Fig. 6.**
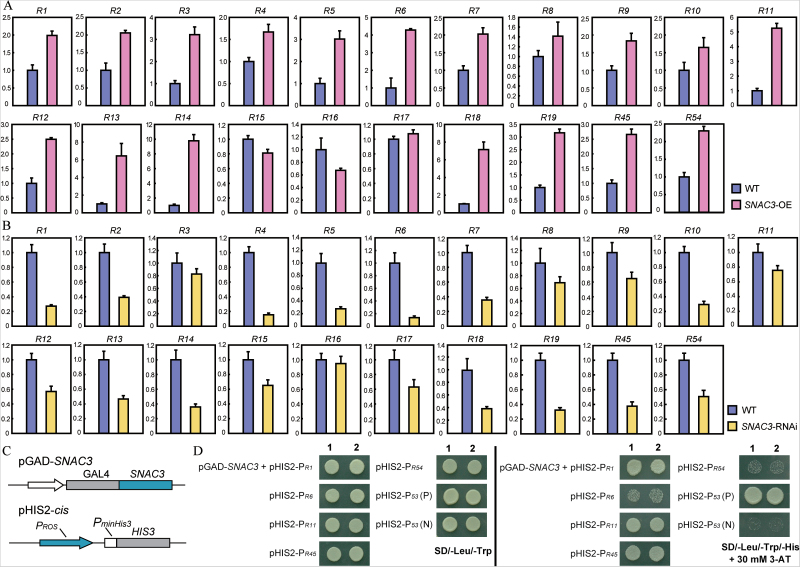
SNAC3 directly regulates ROS-associated genes. (A) Expression of ROS-associated genes in *SNAC3*-overexpression materials. Error bars indicated the SE based on three technical replicates. (B) Expression of ROS-associated genes in *SNAC3*-repression materials. Error bars indicate the SE based on three technical replicates. (C) The schematic structure of the constructs for yeast one-hybrid analysis. (D) pGAD-*SNAC3* and each of the reporter constructs were co-transformed into yeast strain Y187 and the transformants were examined by growth performance on SD/-Leu/-Trp medium and on SD/-Leu/-Trp/-His medium containing 30 mmol l^–1^ 3-AT. pGAD-*53* was co-transformed with pHIS2-P_*53*_ as a positive control (P), and pGAD-*SNAC3* was co-transformed with pHIS2-P_*53*_ as a negative control (N). Labels 1 and 2 indicate two independent transformants of each transformation event. (This figure is available in colour at *JXB* online.)

The positive effect of *SNAC3*-OE on oxidative stress tolerance hinted that SNAC3 may be involved in the regulation of ROS homeostasis. ROS-scavenging enzymes play crucial roles in ROS homeostasis, and ascorbate peroxidase (APX), superoxide dismutase (SOD), and catalase (CAT) are three major types of these enzymes (Apel and Hirt, 2004). Therefore, the expression levels of 19 genes (named R1–R19 in this study) encoding APX, SOD, or CAT were detected in the *SNAC3*-OE and RNAi transgenic plants along with the WT by using qPCR. Two other ROS-related genes, R45 (*RbohF*, LOC_Os08g35210) and R54 (*Prx IIE2*, LOC_Os02g09940), with significant co-expression with *SNAC3* in the microarray database were also included in the qPCR analysis. The results indicated that 14 of the 19 tested ROS-related genes were dramatically up-regulated in the *SNAC3*-OE plants (Fig. 6A). In contrast, the transcript levels of 13 genes were significantly decreased in the *SNAC3*-RNAi plants (Fig. 6B). The above results suggested that SNAC3 may be a key regulator upstream of a large number of ROS genes, and overexpressing *SNAC3* could trigger the induction of a series of ROS genes to cope positively with oxidative stress and other adverse environmental conditions.

### Identification of putative targets of SNAC3

Since so many genes which were involved in ROS pathways were up-regulated in *SNAC3*-OE transgenic plants, and the *SNAC3*-OE plants accumulated less H_2_O_2_ under heat stress conditions, it was presumed that some of these genes might be directly regulated by SNAC3. Five ROS-related genes showing significant co-expression patterns with *SNAC3* (Supplementary Table S2 at *JXB* online) were identified by using the publicly available rice expression profile databases (CREP, http://crep.ncpgr.cn/crep-cgi/home.pl; and Rice Oligonucleotide Array Database, http://www.ricearray.org/coexpression/coexpression.shtml). The five genes are *R1* (LOC_Os02g02400, *CATA*), *R6* (LOC_Os04g14680, *APX3*), *R11* (LOC_Os02g34810, *APX8*), *R45* (LOC_Os08g35210, NADPH oxidase, *RbohF*), and *R54* (LOC_Os02g09940, peroxiredoxin, *Prx IIE2*), and the corresponding names are shown in Fig. 6. Sequence analysis indicated that the NAC-specific *cis*-elements including NACRS and CDBS are enriched in the promoters of these genes (Supplementary Table S3), which further supported that some of these ROS-related genes may be the direct target genes of SNAC3. Yeast one-hybrid assay was performed to examine whether SNAC3 can bind to the promoters of the five genes mentioned above. Fragments containing the NACRS and CDBS elements were amplified from the promoter regions of five ROS genes and inserted into the pHIS2 vector to generate the destination constructs (pHIS2-P_*R1*_, pHIS2-P_*R6*_, pHIS2-P_*R11*_, pHIS2-P_*R45*_, and pHIS2-P_*R54*_) which were subsequently co-transformed with the pGADT7-*SNAC3* fusion construct into the yeast strain Y187 (Fig. 6C). The growth performance showed that the co-transformants of pGAD-*SNAC3* along with pHIS2-P_*R1*_, pHIS2-P_*R11*_, or pHIS2-P_*R45*_ grew well on the SD/-Trp/-Leu/-His medium in the presence of 30mM 3-AT, whereas the growth of the co-transformants of pGAD-*SNAC3* along with pHIS2-P_*R6*_ and pHIS2-P_*R54*_ was significantly inhibited, similar to the negative control (Fig. 6D), suggesting that SNAC3 could bind to the promoters of *R1* (*CATA*), *R11* (*APX8*), and *R45* (*RbohF*) and activate the reporter gene expression in yeast.

### Identification of SNAC3-interacting proteins

**Fig. 7.**
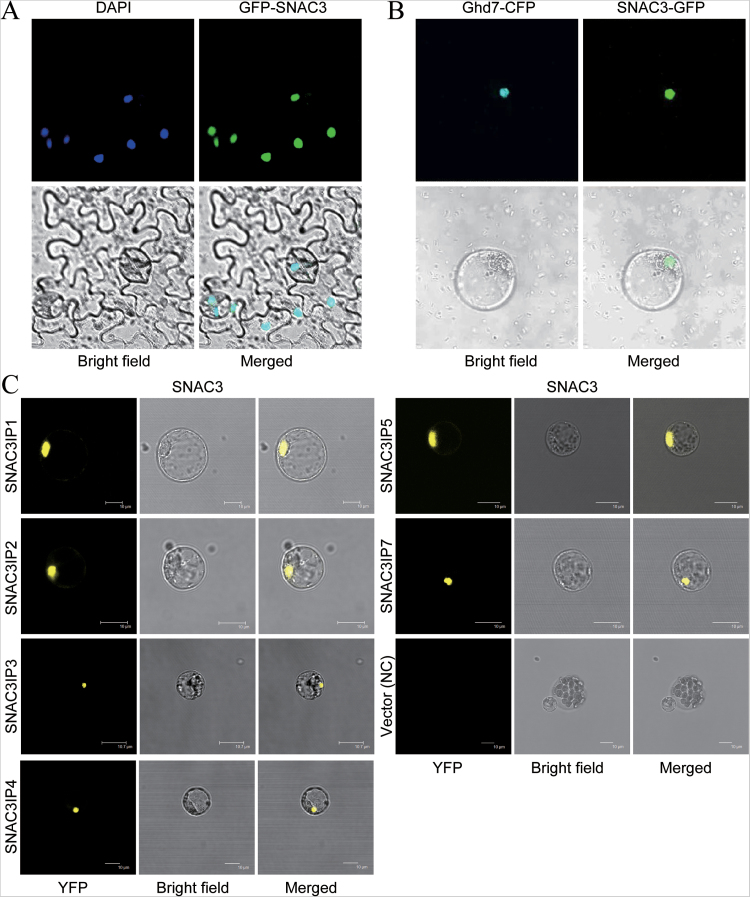
Identification of SNAC3-interacting proteins. (A) Nuclear localization of SNAC3 in tobacco epidermal cells. Infection of tobacco leaves by *Agrobacterium* containing the *35S* promoter-driven SNAC3–GFP fusion expression construct. DAPI staining indicates the nuclear regions. (B) Nuclear localization of SNAC3 in rice protoplasts. Ghd7–CFP and SNAC3–GFP were co-transformed into etiolated shoot protoplasts of rice. Ghd7–CFP was used as a nuclear marker. (C) Confirmation of SNAC3 and SNAC3IP1/SNAC3IP2/SNAC3IP3/SNAC3IP4/SNAC3IP5/SNAC3IP7 interaction using BiFC in rice protoplasts. NC, negative control.

To verify SNAC3 as a transcription factor and identify the putative interacting protein of SNAC3, the subcellular location of the SNAC3 protein was first determined. A construct expressing a *GFP*-*SNAC3* fusion gene driven by the *Cauliflower mosaic virus* 35S (*CaMV35S*) promoter was generated and introduced into tobacco using an *Agrobacterium*-mediated method to perform a transient expression in the leaves. As shown in Fig. 7A, a green fluorescent proteim (GFP) fluorescence signal was detected in the region which overlapped with the nucleus-specific DAPI staining, suggesting that SNAC3 is a nuclear protein. Transient expression in a rice protoplast system was applied to confirm this result. A Ghd7–cyan fluorescent protein (CFP) fusion protein was used as a nuclear localization marker, as Ghd7 is a previously reported nuclear protein in rice (Xue *et al.*, 2008). A SNAC3–GFP fusion protein was co-expressed with Ghd7–CFP in rice protoplasts, and the fluorescence signal was observed and captured using confocal microscopy. The results revealed that the GFP fluorescence signal matched exactly with the CFP fluorescence signal (Fig. 7B), suggesting that SNAC3 is located in the nucleus.

By using the SNAC3 regulatory region as the bait for yeast two-hybrid screening, eight putative SNAC3-interacting proteins were obtained (Supplementary Table S4 at *JXB* online). The interactions between SNAC3 and six interacting proteins (SNAC3IP1, SNAC3IP2, SNAC3IP3, SNAC3IP4, SNAC3IP5, and SNAC3IP7) were confirmed by BiFC using the rice protoplast expression system (Fig. 7C). The putative SNAC3-interacting proteins, including phosphoglycerate mutase, WD domain-containing protein, cytochrome P450 72A1, protein phosphatase 2C, and oxidoreductase, have been reported to be associated with plant responses to abiotic stress (Tahtiharju and Palva, 2001; Bray, 2004; Lee *et al.*, 2010; Kosova *et al.*, 2011; Narsai *et al.*, 2013).

### *SNAC3* function may be independent of ABA

**Fig. 8.**
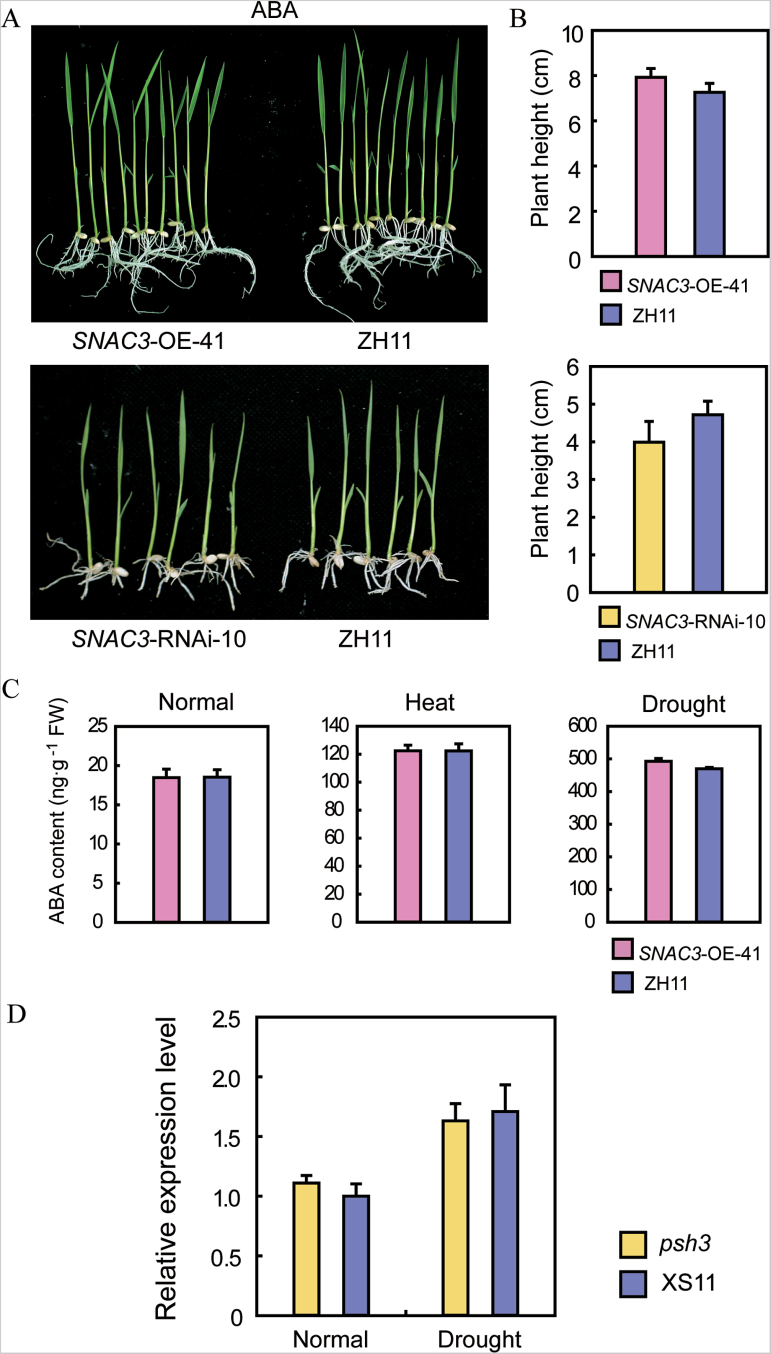
*SNAC3* regulates stress responses in an ABA-independent manner. (A) No significant difference was observed between the *SNAC3*-OE, *SNAC3*-RNAi, and the control (ZH11) under ABA treatment. (B) Plant height of *SNAC3*-OE, *SNAC3*-RNAi, and ZH11 under ABA treatment. (C) Endogenous ABA content of *SNAC3*-OE and ZH11 WT leaves under normal conditions, drought stress, and heat stress conditions. Data represent the mean ±SE (*n*=3). (D) Expression analysis of *SNAC3* in the ABA-deficient mutant *phs3* under normal and drought stress conditions. Error bars indicate the SE based on three replicates.

As an essential phytohormone, ABA controls various processes throughout the life cycle of plants, especially in response to external environmental stimuli (Schroeder *et al.*, 2001). Previous studies have reported that several NAC genes are involved in stress responses in an ABA-dependent manner. To examine whether the function of *SNAC3* in stress resistance relies on ABA, the sensitivity of *SNAC3*-OE and RNAi transgenic plants to ABA treatment was tested at the post-germination stage. Compared with the WT, neither the *SNAC3*-OE nor RNAi plants showed significant differences in shoot lengths under ABA treatment (Fig. 8A, B). Determination of the endogenous ABA content indicated that there was no significant difference in the ABA content between *SNAC3*-OE and the control plants under both normal and stress (heat or drought) conditions (Fig. 8C). The transcript levels of *SNAC3* in the ABA-deficient mutant *phs3* and the corresponding WT (XS11) were also analysed by qPCR. As shown in Fig. 8D, the expression levels of *SNAC3* showed no obvious differences in the *phs3* mutant and in the XS11 WT. Furthermore, the transcript abundance of a series of key genes for ABA biosynthesis and signal transduction showed no significant differences in the *SNAC3*-OE and WT plants (data not shown). These results suggest that *SNAC3* may function mainly through ABA-independent pathways.

## Discussion

### SNAC3 confers heat tolerance by modulating the expression of downstream ROS genes

Various stresses including drought, high salinity, extreme temperatures, and heavy metals may give rise to the overaccumulation of ROS, which can cause damage to plants (Mittler, 2002). Two NAC transcription factors (NTL4 and JUB1) were demonstrated to be involved in abiotic stress responses through the regulation of ROS metabolism (Lee *et al.*, 2012; Wu *et al.*, 2012). However, NAC transcription factors conferring stress resistance by directly regulating ROS metabolism genes have not been reported. The present data revealed that SNAC3 is a positive regulator in response to heat and oxidative stresses in rice. The H_2_O_2_ and MDA content which accumulated in the leaves of transgenic plants overexpressing *SNAC3* was significantly lower than that in the control plants, and the relative ion leakage was also lower in the transgenic plants (Fig. 2C, D), suggesting that the improved heat resistance of the *SNAC3*-OE plants may be due to the stronger capability to scavenge ROS under heat stress conditions to maintain a lower degree of membrane lipid peroxidation. Supporting this, overexpression of *SNAC3* led to the induction of many ROS-associated genes, while suppression of *SNAC3* caused the down-regulation of these genes (Fig. 6A, B). Therefore, it is proposed that SNAC3 is a positive NAC regulator of heat resistance through controlling the expression of downstream genes involved in the ROS pathway.

The results indicated that *SNAC3* overexpression not only enhanced heat and oxidative tolerance, but also improved the drought resistance of the transgenic rice plants. However, distinct from the up-regulated ROS-scavenging genes and reduced accumulation of H_2_O_2_ in the *SNAC3*-OE plants under heat stress, there were no significant differences in H_2_O_2_ accumulation between the *SNAC3*-OE and the control plants under drought stress conditions (Fig. 5A, B), implying that *SNAC3* may confer heat and drought stress tolerance via different pathways. Measurements on the rates of water loss revealed that *SNAC3*-OE plants lose water more slowly than the control plants (Fig. 3C). Additionally, overexpressing *SNAC3* increased the osmotic tolerance to mannitol (Fig. 3F). These results imply that *SNAC3* may regulate drought resistance mainly by reducing water loss in leaves and osmotic adjustment through unknown pathways.

Inadequate water supply is often accompanied by high temperature, which severely limits the yield of crops. Therefore, it is important to pyramid the genes which can increase tolerance to drought or heat stresses or to discover genes which can enhance tolerance to both drought and heat stresses (Barnabás *et al.*, 2002). Overexpressing *SNAC3* can increase not only heat tolerance via enhancing the cell membrane stability and maintaining the redox homeostasis, but also drought tolerance by reducing water loss in rice. Therefore, this gene may be a promising candidate gene for genetic engineering in generating crops with improved drought and heat tolerance.

### Target genes of SNAC3 and their potential roles in stress response

Transcription factors and *cis*-acting elements are central components of the regulation networks in plants. Identification of the direct downstream target genes is an effective strategy to elucidate the function of the SNAC3 transcription factor. Co-expression analysis indicated that five ROS-scavenging or metabolism-related genes, namely *R1* (LOC_Os02g02400, *CATA*), *R6* (LOC_Os04g14680, *APX3*), *R11* (LOC_Os02g34810, *APX8*), *R45* (LOC_Os08g35210, *RbohF*), and *R54* (LOC_Os02g09940, *Prx IIE2*), shared significant correlations with the *SNAC3* expression levels under normal or stress conditions. The NAC-specific NACRS and CDBS *cis*-elements are enriched in their promoter regions (Supplementary Table S3 at *JXB* online), and these genes were up-regulated in *SNAC3*-OE plants (Fig. 6A). Yeast one-hybrid analysis further substantiated that SNAC3 can bind to the promoter regions of three of the genes (*R1*, *R11*, and *R45*). *R1*, *R11*, and *R45* encode OsCATA, OsAPX8, and OsRboh F, respectively, and they are all responsive to oxidative stress. Homologous genes of *R1*, *R11*, and *R45* in *Arabidopsis* encode CAT2 (At4g35090), thylAPX (At1g77490), and NADPH oxidase F (At1g64060, AtrbohF), respectively, and their participation in the maintenance of the intracellular redox equilibrium and ROS signalling transduction has been well documented (Kalbina and Strid, 2006; Queval *et al.*, 2007; Maruta *et al.*, 2010; Mittler *et al.*, 2011; Wang *et al.*, 2013). It is speculated that R1, R11, and R45 probably have similar functions in rice, and *SNAC3* confers heat resistance mainly through the control of the expression of these ROS-related target genes. CAT has been regarded as in charge of removing excess reactive oxygen intermediates (ROIs) during stress, while APX is responsible for fine-tuning of ROS signals (Mittler, 2002). As key producers of ROS under both normal and stress conditions in plants, NADPH oxidases (Rboh/Nox) are considered as the engines of ROS signalling and involved in response to stress (Suzuki *et al.*, 2013; Wang *et al.*, 2013). Rbohs not only regulate a multitude of vital biological processes, but also act as pivotal signalling nodes in the ROS network and integrate a great variety of signal transduction pathways with ROS signalling (Suzuki *et al.*, 2013). *OsNox6* expression was found to be slightly down-regulated by drought stress and up-regulated by high temperature (Wang *et al.*, 2013). The interaction between SNAC3 protein and the promoter regions of three ROS-associated genes (*R1*-*CATA*, *R11*-*APX3*, and *R45*-*RbohF*) revealed that SNAC3 is likely to regulate not only ROS scavenging but also ROS metabolism.

Six SNAC3-interacting protein genes with evidence for a role in stress responses have been identified here. Further investigations are needed to illustrate the detailed mechanisms of the interactions between SNAC3 and these proteins for regulating stress responses in rice.

### *SNAC3* mediated stress responses in an ABA-independent manner

The phytohormone ABA co-ordinates a complicated network in response to various abiotic stress conditions in plants, and numerous transcription factors take part in stress responses in ABA-mediated pathways. Many stress-responsive genes contain an ABA-responsive promoter element (ABRE) recognition sequence with an ACGT core motif. Basic leucine zipper transcription factors called ABFs/AREBs such as ABF2 and AREB1 are documented to induce stress-responsive gene expression by interacting with the corresponding ABRE *cis*-acting elements (Xiong *et al.*, 2002; Kim *et al.*, 2004; Fujita *et al.*, 2005). Other transcription factors including CBF4 (DREB1D), RD22BP1, and AtMYB2 are also reported to be regulated through ABA-dependent pathways (Abe *et al.*, 1997, 2003; Haake *et al.*, 2002). Several NAC transcription factors have been reported to play critical roles in the ABA-mediated stress response pathways in plants. In contrast, the present data indicated that *SNAC3* may exert its functions in stress resistance mainly through ABA-independent pathways. Compared with the WT, neither the *SNAC3*-OE nor the RNAi transgenic plants exhibited obvious changes in the sensitivity to ABA treatment (Fig. 8A, B). The *SNAC3*-OE and the control plants had equivalent endogenous ABA levels under heat, drought, and normal conditions (Fig. 8C). The expression level of *SNAC3* was not affected in the ABA-deficient mutant in rice (Fig. 8D). In addition, overexpression of *SNAC3* in rice did not alter the transcript abundance of the genes which are essential for ABA biosynthesis and the signal transduction pathways (data not shown). Together, this evidence supported that *SNAC3* mediates drought and heat stress resistance mainly in an ABA-independent manner.

In conclusion, SNAC3 is a stress-responsive NAC transcription factor which confers heat tolerance by means of modulating H_2_O_2_ homeostasis through controlling the expression of ROS genes. *SNAC3* also contributes to drought resistance and osmotic adjustment. In contrast to some previously reported NAC genes, *SNAC3* may function mainly through ABA-independent pathways in rice. The positive effect of *SNAC3* in improving resistance to multiple stresses implies high potential in engineering this gene for stress resistance improvement in crops.

## Supplementary data

Supplemenaty data are available at *JXB* online.

Figure S1. Schematic diagram of *SNAC3* gene structure.

Figure S2. Overexpression of *SNAC3*.

Figure S3. Suppression of *SNAC3* by RNAi.

Table S1. List of primers used in this study.

Table S2. Correlation between *SNAC3* and the ROS genes.

Table S3. Distribution of NACRS and CDBS elements in the ROS genes.

Table S4. Information on putative SNAC3-interacting proteins.

Supplementary Data

## Abbreviations:

DAB: 3,3′-diaminobenzidine
MDA: malondiadehyde
MV: methyl viologen
NAC: NAM, ATAF1/2, and CUC2
OE: overexpression
qPCR: quantitative real-time reverse transcription–PCR
ROS: reactive oxygen species.
