# Supplementary material for: A stress-responsive NAC transcription factor SNAC3 confers heat and drought tolerance through modulation of reactive oxygen species in rice
Source: J Exp Bot. 2015 Aug 10;66(21):6803–17. doi: 10.1093/jxb/erv386 (PMC4623689; doi:10.1093/jxb/erv386)
Supplement: Supplementary Data [file supp_66_21_6803__index.html]

A stress-responsive NAC transcription factor SNAC3 confers heat and drought tolerance through modulation of reactive oxygen species in rice — A stress-responsive NAC transcription factor SNAC3 confers heat and drought tolerance through modulation of reactive oxygen species in rice — Supplementary Data 

# A stress-responsive NAC transcription factor SNAC3 confers heat and drought tolerance through modulation of reactive oxygen species in rice

## Supplementary Data

Data files

- Supplementary Data - Supplementary Data
