# Supplementary material for: A stress-responsive NAC transcription factor SNAC3 confers heat and drought tolerance through modulation of reactive oxygen species in rice
Source: J Exp Bot. 2015 Aug 10;66(21):6803–17. doi: 10.1093/jxb/erv386 (PMC4623689; doi:10.1093/jxb/erv386)
Supplement: Supplementary Data [file supp_erv386_jexbot154096_file001.pdf]

Supplementary Table S1. List of primers used in this study.

Purpose	Gene	TIGR locus ID	Sense primer (5'→3')	Anti-sense primer (5'→3')
OE construct	*SNAC3*	LOC_Os01g09550	TTggtaccCTCGTCCTCTTTGCTCT	TTggatccCTTCTCCCAACCGTAGT
RNAi construct	*SNAC3*	LOC_Os01g09550	TCGactagtggtaccTGCCAGCAATCCTTCT	TTAgagctcggatccCCCAACCGTAGTACATACA
Promoter construct	*SNAC3*	LOC_Os01g09550	AAgtcgacCTGGAAACCGTGAGAC	AAgtcgacGCTGCTTTCACAAGTTTA
Subcellular location	*SNAC3* (pH7WGF2.0)	LOC_Os01g09550	*att*B1-TGAATAGGGGGCACATCA	*att*B2-TGGTTAGTTTTCTTTTGGTATTG
constructs	*SNAC3* (HBT)	LOC_Os01g09550	TTtctagaATGAATAGGGGGCACAT	TTtctagaTCCATGATGATCCTGGTT
QPCR	*SNAC3*	LOC_Os01g09550	GCACGTCTTCGAGCACAAAA	TGGCTGGTAAGGCCATTCTG
	R1 (CATA)	LOC_Os02g02400	CCCCAAGGTCTCCCCTGA	AACGACTCATCACACTGGGAGAG
	R2 (CATB)	LOC_Os06g51150	GCTGGTGAGAGATACCGGTCA	TCAACCCACCGCTGGAGA
	R3 (CATC)	LOC_Os03g03910	CGAAGCCGAGCATGTAAGGA	GGCTGAGGCCTTCCGTG
	R4 (APX1)	LOC_Os03g17690	TCCACCCAGGAAGGGAGG	TTGGTAGCATCAGGAAGACGG
	R5 (APX2)	LOC_Os07g49400	TCCCCTACCCTGCTGCATC	ACCAGCCAACCACTCGCA
	R6 (APX3)	LOC_Os04g14680	AGCACTCTCAGGCGGCC	AGGCACCATCAAATCCTGATCT
	R7 (APX4)	LOC_Os08g43560	CGGCCCTGCATCTACGAA	CAGCTACCCCGACAGCACTC
	R8 (APX5)	LOC_Os12g07830	CCGGTGGCCCCAAGAT	GGTGGGCATTCTTCACCATC
	R9 (APX6)	LOC_Os12g07820	CCCCAAGATCCCCATGATCTA	CCTCTGGCGGGCATTG
	R10 (APX7)	LOC_Os04g35520	TTCACGTTGGACGGTTAATGC	TTTCTGTAAAAGTGGTTGGCCA
	R11 (APX8)	LOC_Os02g34810	ATCATCGCCAGCGGATGA	GCAGCGACGAAGGGCTC
	R12 (putative copper/ zinc superoxide dismutase)	LOC_Os03g11960	CAGATTTCACTAAGCGGGCC	CTTCCTAGGTCATCAGAATCAGCA
	R13 (SodCc1)	LOC_Os03g22810	TGCGCATGGTTTTTGGTG	CATAGTTCATTGGGCGCCA
	R14 (putative copper chaperone for superoxide dismutase)	LOC_Os04g48410	AGATCAGATCCTGGCATTGCA	TTCTTGTAGTTCTCGCCAACCC

Purpose	Gene	TIGR locus ID	Sense primer (5'→3')	Anti-sense primer (5'→3')
QPCR	*R15* (SodA1)	LOC_Os05g25850	CCGCCATCGTGCACCT	TCGAATGATTGACATGGCCTC
	R16 (putative superoxide dismutase)	LOC_Os06g02500	CGGAGCCAAGTAAGCTACGG	TGTTACAAGTCCATCATCCCCA
	R17 (Fe-SOD)	LOC_Os06g05110	CGACGCCGAGGAATTTCTAG	AGGTGGTGTAAGTGTCTCTCATGC
	R18 (SodCc2)	LOC_Os07g46990	ATTCCATGTGCACGCGC	GGATTGAAGTGTGGTCCAGTTG
	R19 (plastidic Cu/Zn-SOD)	LOC_Os08g44770	TGTGACGGGACTTACTCCTGG	CACCCATTCGTAGTATCGCCA
	R45 (RbohF)	LOC_Os08g35210	GCCCTCAGCCATCGTCTATC	CCATATATAGTATCACTGCGCAATGC
	R54 (Prx IIE2)	LOC_Os02g09940	GCGCTCTGAAGATGGAATCC	TCACAACGACGACGCTACTCA
	Ubiquitin	LOC_Os03g13170	AACCAGCTGAGGCCCAAGA	ACGATTGATTTAACCAGTCCATGA
Yeast one-hybrid constructs	*SNAC3*	LOC_Os01g09550	CAGgaattcGGCATGAATAGGGGGCAC	CAGggatccTGGTTAGTTTTCTTTTGGTATTG
	R1 (CATA)	LOC_Os02g02400	ATAgaattcATAAACGAGGGTGTAGC	ATAgagctcGAGGTGGTGAGGAGCA
	R6 (APX3)	LOC_Os04g14680	ATAgaattcGCCGAGTAGGGCTGAA	ATAgagctcGGCAAGGGATGTCTCC
	R11 (APX8)	LOC_Os12g07830	ATAgaattcCATCGGTGGCTAACGG	ATAgagctcGAAGGTTTCGGCAAGG
	R45 (RbohF)	LOC_Os08g35210	ATAgaattcCCTGCTTTGGTAATGG	ATAgagctcCAAGAGGACGGAGATA
	R54 (Prx IIE2)	LOC_Os02g09940	ATAgaattcGGAGCAGCCAGAGGTCA	ATAgagctcCCGCAAGGGCGATTTT
BiFC assay	*SNAC3*	LOC_Os01g09550	AGTggatccATGAATAGGGGGCACATC	GAGggtaccTCCATGATGATCCTGGTTG
	SNAC3IP1	LOC_Os03g01180	CCactagtAGACGCTGCTGGAGTAG	GAgtcgacGTCGGGGACCTCAGATG
	SNAC3IP2	LOC_Os01g63900	AGTggatccTTCTTCGAGCAGGTGAG	GAGggtaccTTTCTTGAAAAGTCTGTTC
	SNAC3IP3	LOC_Os01g72880	AGTggatccACCCTCGACGGAACCGC	CTgtcgacTCCTGATTCCTGGGGCG
	SNAC3IP4	LOC_Os05g33600	AGTggatccGAGTGAGTCCCGACCA	GAGggtaccACACCTTTCAAAAATCTTG
	SNAC3IP5	LOC_Os06g39600	AGTggatccGCCAATCCTACCACATCC	GAGggtaccGTTGCCACCAGACTCCT
	SNAC3IP6	LOC_Os04g52870	AGTggatccAAATCGGAACCCTAACC	GAGggtaccATCAGCCAAGCTATCAAGA
	SNAC3IP7	LOC_Os10g27340	CCactagtAGGCTGCTCCCATCCTC	GACctcgagTTTCTTCTTGGCGGCAG
	SNAC3IP8	LOC_Os02g43930	CCactagtGGTCGCTTCTTCCTCTT	GAGggtaccCTGCTGGGCACACTGTA

Supplementary Table S2. Correlation between *SNAC3* and the ROS genes.

SNAC3	Normal condition	Abiotic stress	Biotic stress
R1		√（0.5022）	√（0.6330）
R6	√（0.5076）		√（0.6604）
R11			
R45	√（0.5434）		√（0.5404）
R54	√（0.5076）		

Supplementary Table S3. Distribution of NACRS and CDBS elements in the ROS genes.

Promoter	Fragments length	NACRS number	CDBS number
R1	498 bp	2	2
R6	913 bp	3	3
R11	820 bp	1	8
R45	1376 bp	2	5
R54	1525 bp	1	17

Supplementary Table S4. Information of putative SNAC3-interacting proteins.

Name	TIGR locus ID	Annotation
SNAC3IP1	LOC_Os03g01180	Phosphoglycerate mutase
SNAC3IP2	LOC_Os01g63900	WD domain, G-beta repeat domain containing protein
SNAC3IP3	LOC_Os01g72880	DNA mismatch repair protein MLH1
SNAC3IP4	LOC_Os05g33600	Cytochrome P450 72A1
SNAC3IP5	LOC_Os06g39600	Protein phosphatase 2C
SNAC3IP6	LOC_Os04g52870	OsFBW1, F-box domain and WD40 repeat containing protein
SNAC3IP7	LOC_Os10g27340	Oxidoreductase
SNAC3IP8	LOC_Os02g43930	Chaperone protein dnaJ

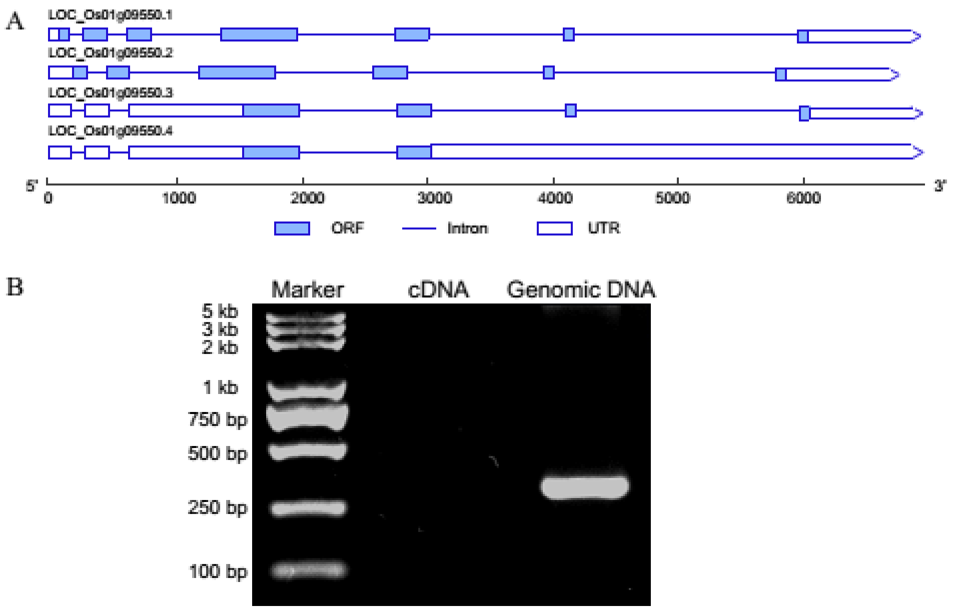

Supplementary Figure S1 Schematic diagram of *SNAC3* gene structure. (A) Putative transcripts of *SNAC3*. (B) N-terminus PCR of LOC_Os01g09550.1. “cDNA” indicated PCR from the cDNA template, and “Genomic DNA” indicated PCR from the genomic DNA template.

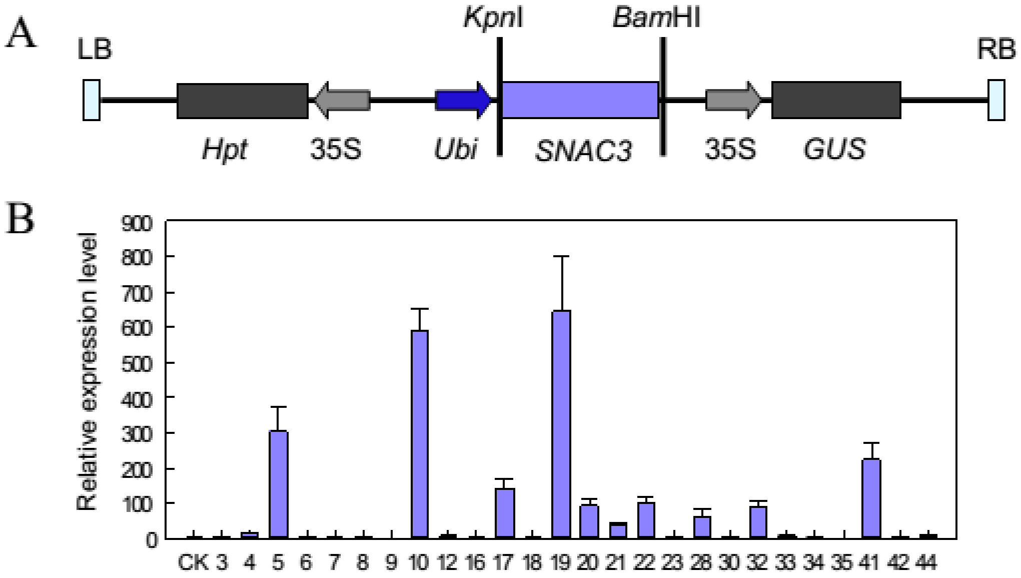

Supplemental Figure S2 Overexpression of *SNAC3*. (A) Schematic diagram of *SNAC3* overexpression construct. (B) Expression level analysis of *SNAC3* in transgenic plants by qPCR.

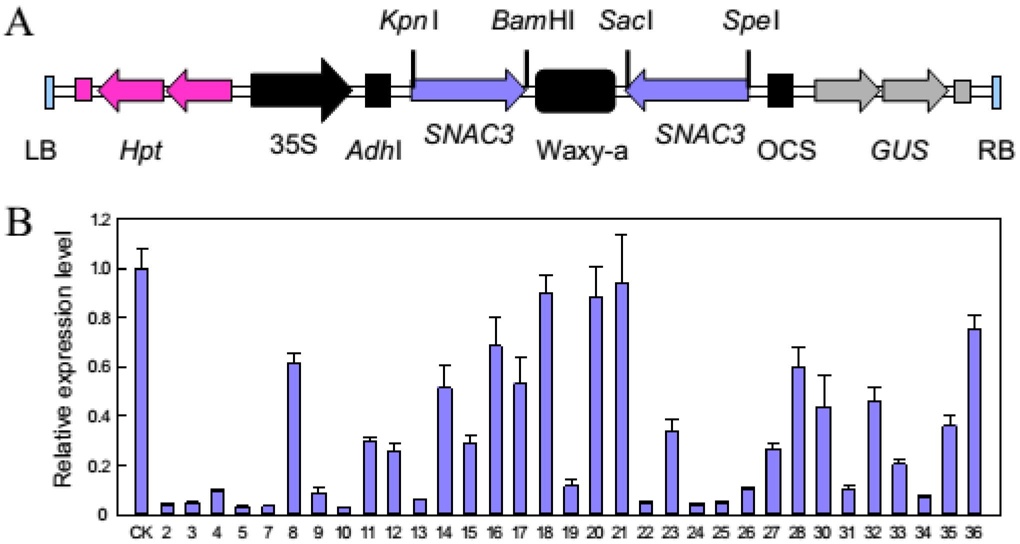

Supplementary Figure S3 Supression of *SNAC3* by RNAi. (A) Schematic diagram of *SNAC3* RNAi construct. (B) Expression level analysis of *SNAC3* in RNAi transgenic plants by qPCR.
